# Evaluation of burnout among stay-behind healthcare workers during the current Omicron wave of COVID-19 in Taizhou, China

**DOI:** 10.3389/fpsyt.2022.1022881

**Published:** 2022-10-21

**Authors:** Shuang-Jun Pan, Wei-Yan Qian, Yu-Pei Yang, Mei-Xian Zhang, Xiao-Ming Hu, Hai-Xiao Chen, Tao-Hsin Tung

**Affiliations:** ^1^Department of Neurosurgery, Taizhou Hospital of Zhejiang Province Affiliated to Wenzhou Medical University, Zhejiang, China; ^2^Department of Hematology, Taizhou Hospital of Zhejiang Province Affiliated to Wenzhou Medical University, Zhejiang, China; ^3^Evidence-based Medicine Center, Taizhou Hospital of Zhejiang Province Affiliated to Wenzhou Medical University, Zhejiang, China; ^4^Department of Orthopedics, Taizhou Hospital of Zhejiang Province Affiliated to Wenzhou Medical University, Zhejiang, China

**Keywords:** burnout, COVID-19, stay-behind, healthcare, workers

## Abstract

**Background:**

Since February 2022, a new Omicron wave of COVID-19 emerged in Shanghai, China. Many healthcare workers came to Shanghai from hospitals of other parts of China as aid workers. Hospitals in areas with mild COVID-19 outbreaks will inevitably be understaffed, it is likely to cause job burnout of stay-behind healthcare workers. Stay-behind healthcare workers were those who had not been dispatched to support COVID-19 prevention and control in other regions. This study was designed to evaluate the burnout among stay-behind healthcare workers in the current COVID-19 Omicron wave in Taizhou, China.

**Methods:**

A population-based, anonymous, cross-sectional online survey was designed in the Wen-Juan Xing platform. The survey was sent to all stay-behind healthcare workers of the hospital (*n* = 1739) from April 29 to May 3, 2022. The Maslach Burnout Inventory-General Survey (MBI-GS) was used for the burnout survey. For univariate analysis, the χ^2^ test and one way ANOVA were used to assess differences in categorical variables and continuous variables, respectively. The effect of independent associated risk factors on each type of burnout was examined using the multinomial logistic regression model.

**Results:**

A total of 434 participants completed the survey invitation effectively. A total of 71.2% of stay-behind healthcare workers experienced burnout during COVID-19, including 54.8% experiencing mild to moderate burnout and 16.4% experiencing severe burnout. Night shift, depression, social support, positive coping and number of children appeared to be significantly related to mild to moderate burnout. Night shift, depression, social support, positive coping, number of children, professional title, and anxiety appeared to be significantly related to severe burnout.

**Conclusion:**

Job burnout among stay-behind healthcare workers was an important problem during the current Omicron wave of COVID-19. Night shift, depression, social support, positive coping, and number of children were associated with mild to moderate and severe burnout. Anxiety and professional title were associated with severe burnout.

## Introduction

Job burnout refers to a comprehensive symptom of excessive physical and mental consumption and energy exhaustion caused by long-term work pressure (1, 2). Medical staff experience high work intensity, heavy social responsibility, high occupational risk, and high incidence of occupational burnout (3). Healthcare workers’ burnout has been reported to be associated with the failure to rescue (4), low patient care quality (5), and job dissatisfaction (6). Job burnout affects the physical and mental health of medical staff (1), and the quality of medical and health services (3).

Since February 2022, a new Omicron wave of COVID-19 emerged in Shanghai, China. There were 601,942 novel COVID-19 cases and 503 deaths by May 4, 2022 (7). The unprecedented efforts of healthcare workers from Shanghai and other cities, efforts of other frontline workers and a comprehensive strategy to combat COVID-19 has led to considerable results (8). Healthcare workers who came to Shanghai from other parts of China are playing an important role in this fight.

However, hospitals in areas with mild COVID-19 outbreaks will inevitably be understaffed because of the dispatching of medical personnel. As sufficient replacement personnel cannot be recruited in a short period, the work originally undertaken by the dispatching medical personnel has to be performed by the stay-behind healthcare workers. The sudden increase in their workload is a dual physical and psychological challenge, which is likely to cause job burnout (1, 9). Studies have shown that workload (9), age (10), family income (10), the frequency of night shifts (11), emotional problems such as anxiety and depression (12, 13), coping style (14), having physical diseases (10) were considered risk factors for job burnout among healthcare workers.

At present, there is no research on burnout among stay-behind healthcare workers during COVID-19, and this study aims to fill this gap in research.

In this study, stay-behind healthcare workers in Taizhou, Zhejiang province were selected as the research subjects to explore the prevalence of burnout during the current Omicron wave of COVID-19 and identify the individual and job-related determinants of burnout, along with COVID-related factors.

## Materials and methods

### Study design and participants

A population-based, anonymous, cross-sectional online survey was designed in the WeChat-incorporated Wen-Juan Xing platform (Changsha Ranxing Information Technology Co., Ltd., Hunan, China). The participants were healthcare workers at a hospital in Taizhou, China. Data was collected from April 29 to May 3, 2022. The survey was sent to all stay-behind healthcare workers of the hospital (*n* = 1,739). A total of 434 participants completed the survey invitation effectively, with a response rate of 25.0%. This study exempted informed consent and was approved by the Ethics Committee of Taizhou Hospital, Zhejiang Province, China (Approval number: K20220410). All procedures were conducted according to the guidelines of our institutional ethics committee and the principles of the Declaration of Helsinki. We performed a logical check of data, excluding those who answered within 200 s. Information about all participants was stored anonymously.

### Measurement instruments

#### Demographic data

This survey included general demographic data and one questionnaire. The questionnaire included 48 items divided into six parts: general situation, job burnout, anxiety, depression, social support, and coping style. The general information included gender, age, working years, department, marital status, education level, professional title, number of children, living conditions of parents, working position, night shifts, and willingness to fight COVID-19. We converted some continuous variables into categorical variables for the convenience of analysis. We divided working years into two categories: < 10 years and ≥ 10 years; number of children into three categories: 0, 1, and ≥ 2; job position into two categories: first and second level, third and fourth level.

#### Maslach Burnout Inventory-General Survey

Burnout was measured by the Maslach Burnout Inventory-General Survey (MBI-GS) (2), which was previously translated into Chinese and has shown good reliability and validity in a Chinese sample. The scale included 15 items in three dimensions of emotional exhaustion (EE), depersonalization (DP) and reduced personal accomplishment (PA), each with 7-point Likert-type, frequency response scale (0 = never, 1 = a few times a year or less, 2 = once a month or less, 3 = a few times a month, 4 = once a week, 5 = a few times a week, 6 = every day) (2). Burnout score = [0.4 × EE + 0.3 × DP + 0.3 × (6–PA)], < 1.5 points indicates no burnout, 1.5–3.5 points indicate mild to moderate burnout, ≥ 3.5 points indicates high burnout (15). The overall Cronbach’s α coefficient of the scale was 0.859, and the Cronbach’s α coefficient of each dimension ranged from 0.872 to 0.944, indicating that the internal consistency reliability of the scale was good (16).

#### Self-rating anxiety scale

The scale was compiled by professor of Duke University in the United States and contains 20 items (17). Likert grade 4 scoring method was used for each item. The scores of all items in the 20 items were added up to obtain the total score, and then the total score was multiplied by 1.25 to obtain the integral part, which is the standard score. Standard score < 50, no anxiety; 50–59 are classified as mild anxiety; 60 ∼ 69 were divided into moderate anxiety; A score ≥ 70 is considered as severe anxiety. The Cronbach’s α coefficient is 0.876.

#### Self-rating depression scale

The scale was compiled by professor of Duke University in the United States and contains 20 items (18). Likert grade 4 scoring method was used for each item. The scores of all items in the 20 items were added up to obtain the total score, and then the total score was multiplied by 1.25 to obtain the integral part, which is the standard score. Standard score < 53 was no depression; 53–62 were classified as mild depression; 63–72 were classified as moderate depression; ≥ 73 was classified as severe depression, and the Cronbach’s α coefficient of the scale was 0.896.

#### Measurement of perceived social support

The Perceived Social Support Scale (PSSS), comprising of 12 items scored on a 7-point rating scale (1 = very strongly disagree, 7 = very strongly agree), was used to measure the perceptions of social support received from three sources: family, friends and others (19). The higher the score, the higher the level of social support perceived by an individual. The scale has a good internal reliability (20). The Cronbach’s alpha for PSSS in this study was 0.980.

#### Measurement of coping style

Coping was evaluated by the Simplified Coping Style Questionnaire (SCSQ), which was developed based on the coping styles cognitive theories (21), has been proven to have a good reliability and validity in previous study (22). It consists of 20 items to identify the attitude or potential actions that an individual will adopt when experiencing setbacks or difficulties. Each item was rated on a four-point scale ranging from 0 (never) to 3 (very often). The dimensions of the questionnaire include positive coping style (12 items) and negative coping style (8 items). Higher scores indicate preference of adopting the relevant coping style. The Cronbach’s alpha for this sample was 0.920.

### Statistical analysis

We converted continuous data, such as age and working time, to classified data. For univariate analysis, the χ2 test and one way ANOVA were used to assess differences in categorical variables and continuous variables, respectively. Multinomial logistic regression is the extension of binary logistic regression when the categorical -dependent outcome has more than two levels. This method was also used to provide a set of coefficients for each of the two comparisons of burnout and to investigate the independence of factors associated with the prevalence of burnout. All data were analyzed using IBM SPSS Statistics software (version 26.0; SPSS Inc., Chicago, IL, USA). A *P*-value less than 0.05 was considered statistically significant.

## Results

**FIGURE 1:**
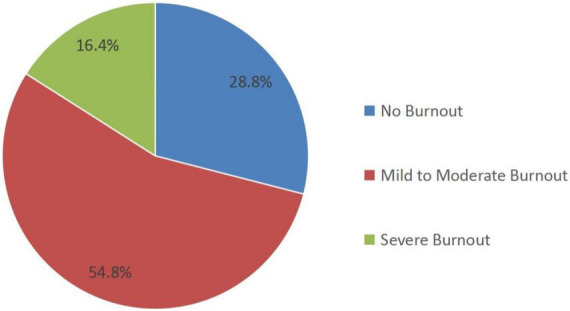
Prevalence of burnout among stay-behind healthcare workers.

A total of 309 (71.2%) of the 434 stay-behind healthcare workers experienced burnout during COVID-19, with 238 (54.8%) reporting mild to moderate burnout, and 71 (16.4%), severe burnout, as shown in Figure 1. The personal and job characteristics of participants are shown in Table 1. It included 374 females and 60 males; the average age was 34.13 ± 7.54 years. Among them, 58.5% had worked for more than 10 years. The highest education level of 85.3% was a bachelor’s degree and above.

**TABLE 1:** Demographic characteristics and burnout among stay behind healthcare workers (*n* = 434).

Variable	Total (*n* = 434)		No burnout (*n* = 125)		Mild to moderate burnout (*n* = 238)		Severe burnout (*n* = 71)		χ^2^	*P*
	*N*	%	*N*	%	*N*	%	*N*	%		
Gender									1.127	0.569
Male	60	13.8	18	30.0	35	58.3	7	11.7		
Female	374	86.2	107	28.6	203	54.3	64	17.1		
Working years									9.055	0.011
<10 years	180	41.5	40	22.2	102	56.7	38	21.1		
≥10 years	254	58.5	85	33.5	136	53.5	33	13.0		
Highest level of education									3.058	0.217
Junior college and below	64	14.7	21	32.8	29	45.3	14	21.9		
Bachelor degree and above	370	85.3	104	28.1	209	56.5	57	15.4		
Marital status									5.785	0.055
Unmarried	129	29.7	29	22.5	72	55.8	28	21.7		
Married	305	70.3	96	31.5	166	54.4	43	14.1		
Number of children									9.687	0.046
0	136	31.3	29	21.3	79	58.1	28	20.6		
1	170	39.2	49	28.8	92	54.1	29	17.1		
≥2	128	29.5	47	36.7	67	52.3	14	10.9		
Living with parents									2.297	0.317
Yes	260	59.9	75	28.8	137	52.7	48	18.5		
No	174	40.1	50	28.7	101	58.0	23	13.2		
Professional title									19.940	0.001
Junior technical title	206	47.5	45	21.8	117	56.8	44	21.4		
Intermediate technical title	142	32.7	48	33.8	70	49.3	24	16.9		
Senior technical title	86	19.8	32	37.2	51	59.3	3	3.5		
Job position									9.916	0.007
First and second level	137	31.6	50	36.5	74	54.0	13	9.5		
Third and fourth level	297	68.4	75	25.3	164	55.2	58	19.5		
Night shift									7.961	0.019
Yes	380	87.6	101	26.6	213	56.1	66	17.4		
No	54	12.4	24	44.4	25	46.3	5	9.3		
Have experience in critical care									1.289	0.525
Yes	252	58.1	73	29.0	142	56.3	37	14.7		
No	182	41.9	52	28.6	96	52.7	34	18.7		
Depression									61.128	<0.001
Yes	157	36.2	12	7.6	102	65.0	43	27.4		
No	277	63.8	113	40.8	136	49.1	28	10.1		
Anxiety									53.877	<0.001
Yes	95	21.9	7	7.4	52	54.7	36	37.9		
No	339	78.1	118	34.8	186	54.9	35	10.3		

	**Mean**	**SD**	**Mean**	**SD**	**Mean**	**SD**	**Mean**	**SD**	**F**	***P***

Age (years)	34.13	7.54	35.48	7.82	34.14	7.46	31.69	6.77	5.852	0.003
Social support	66.27	15.00	75.33	8.79	62.70	15.90	62.31	13.90	37.462	<0.001
Positive coping	2.15	0.63	2.53	0.41	2.00	0.64	1.99	0.62	37.630	<0.001
Negative coping	1.40	0.65	1.30	0.70	1.42	0.61	1.52	0.64	2.772	0.064

Among the participants, 70.3% were married, 39.2% had one child, 29.5% had two or more children, and 59.9% were living with a parent. Regarding professional titles, 47.5% had junior technical, 32.7% had intermediate technical, and 19.8% had senior technical titles. Moreover, 31.6% were in first or second-level positions, indicating that they were the head or deputy head of a medical team and 68.4% were in third or fourth-level positions, indicating that they were important team members. The vast majority (87.6%) were working night shifts.

The prevalent depression was 36.2% (157/434), wherein mild, moderate, and severe were 21.9% (95/434), 13.1% (57/434), and 1.2% (5/434), respectively. The proportion of anxiety was 21.9% (95/434), wherein mild, moderate, and severe accounted for 15.2% (66/434), 4.8% (21/434), and 1.8% (8/434) of participants, respectively.

According to the PSSS, the average social support score for all participants was 66.27 ± 15.00. This score for participants with mild to moderate burnout was 62.70 ± 15.90, with severe burnout was 62.31 ± 13.90, and without burnout was 75.33 ± 8.79. According to the SCSQ, the average positive coping score was 2.15 ± 0.63, this with mild to moderate burnout was 2.00 ± 0.64, with severe burnout was 1.99 ± 0.62, and without burnout was 2.53 ± 0.41. The average negative coping score was 1.40 ± 0.65.

Table 1 also shows that mild to moderate burnout and severe burnout among stay-behind healthcare workers were related to working years (*P* = 0.011), number of children (*P* = 0.046), professional title (*P* = 0.001), job position (*P* = 0.007), night shift (*P* = 0.019), depression (*P* < 0.001), anxiety (*P* < 0.001), age (*P* = 0.003), social support (*P* < 0.001), positive coping style (*P* < 0.001).

The effect of independent associated risk factors on each type of burnout was examined using the multinomial logistic regression model. As presented in Table 2, after adjustment for confounding factors, social support [odds ratio (OR) 0.941, 95% CI 0.910–0.972], positive coping (OR 0.283, 95% CI 0.141–0.565), number of children (1 vs. ≥ 2, OR 1.902, 95% CI 1.00202, 95of children (1 ding vs. no, OR 2.681, 95% CI 1.255–5.714), depression (yes vs. no, OR 3.378, 95% CI 1.527–7.463) appeared to be significantly related to mild to moderate burnout. Social support (OR 0.941, 95% CI 0.905–0.979), positive coping (OR 0.286, 95% CI 0.121 950.941, 95cialof children (1 vs. ≥ 2, OR 4.047, 95% CI 1.594–10.272), professional title (junior vs. senior, OR 11.914, 95% CI 1.787–79.428; intermediate vs. senior, OR 6.452, 95% CI 1.345OR 6.452, 955title (junior vs. no, OR 4.202, 95% CI 1.266–13.889), depression (yes vs. no, OR 4.065, 95% CI 1.508–10.989),anxiety (yes vs. no, OR 7.407, 95% CI 2.387–23.256) appeared to be significantly related to severe burnout.

**TABLE 2:** Multinomial Logistic regression analysis of burnout among stay behind healthcare workers during COVID-19 pandemic (*n* = 434).

Variables	Mild to moderate burnout vs. no burnout			Severe burnout vs. no burnout		
	OR	95%CI	*P*-value	OR	95%CI	*P*-value
Age	0.980	0.913–1.052	0.581	0.958	0.865–1.062	0.414
Social support	0.941	0.910–0.972	<0.001	0.941	0.905–0.979	0.003
Positive coping	0.283	0.141–0.565	<0.001	0.286	0.121–0.678	0.004
Working years						
< 10 vs. ≥ 10	0.712	0.262–1.925	0.503	0.622	0.171–2.266	0.471
Number of children						
0 vs. ≥ 2	1.420	0.552–3.656	0.467	1.495	0.409–5.462	0.543
1 vs. ≥ 2	1.902	1.002–3.609	0.049	4.047	1.594–10.272	0.003
Professional title						
Junior vs. senior	1.851	0.560–6.116	0.313	11.914	1.787–79.428	0.010
Intermediate vs. senior	1.118	0.479–2.609	0.797	6.452	1.345–30.943	0.020
Job position						
First and second level vs. third and fourth level	0.517	0.287–1.136	0.110	0.467	0.171–1.272	0.137
Night shift (yes vs. no)	2.681	1.255–5.714	0.011	4.202	1.266–13.889	0.019
Depression (yes vs. no)	3.378	1.527–7.463	0.003	4.065	1.508–10.989	0.006
Anxiety (yes vs. no)	1.972	0.717–5.405	0.188	7.407	2.387–23.256	0.001

*P*-value for Hosmer-Lemeshow = 0.549.

## Discussion

The study yielded several findings: (1) Due to the serious epidemic in Shanghai, many medical workers have been transferred from different parts of China to help Shanghai during the current Omicron wave of COVID-19 in 2022. As a result, the burnout of stay-behind healthcare workers is moderate. Of the participants, 71.2% suffered from burnout, including 16.4% with severe burnout and 54.8% with mild to moderate burnout. (2) Of the participants, 21.9% suffered from anxiety and 36.2% from depression. (3) Night shift, depression, social support, positive coping, and number of children were associated with mild to moderate burnout. Night shift, depression, anxiety, social support, positive coping, number of children and professional title were associated with severe burnout.

This study found that the prevalence of job burnout among stay-behind healthcare workers in China was 71.2% during the Omicron wave in 2022, which was significantly higher than the prevalence of burnout among frontline medical staff of China during COVID-19 in many previous studies (9, 10, 23, 24). As illustrated in Table 3 (9, 10, 23–32), the prevalence of job burnout varies significantly across different regions and stages of COVID-19, which may be related to the economic and salary levels of different regions, as well as the importance that the public attaches to medical staff and the sense of professional achievement of medical staff. Many previous studies (9, 24) focused on frontline medical staff fighting COVID-19 while ignoring the potential medical risks caused by job burnout of stay-behind medical staff. It is reasonable to infer that with the evolution of COVID-19, many medical staff members had been transferred to the frontline of epidemic prevention and control, leading to a shortage of staff in hospitals. The stay-behind medical staff need to finish their work and that of the dispatched personnel. They have a particularly high incidence of burnout as they struggle to cope with the increased workload caused by the increased complexity of the COVID-19 response strategy.

**TABLE 3:** The estimates of burnout among healthcare workers during COVID-19 pandemic from different cross-sectional studies.

Author	Time of data collection	Sample size	Setting	Study participants	Prevalence of burnout (%)	References
Zhang et al.	2020.2.18–3.4	1,163	China (Wuhan, Harbin, and Shenzhen)	Mixed frontline healthcare workers	48.6%	(9)
Huo et al.	2020.2.14–3.29	606	China (133 cities)	Mixed frontline medical workers	36.5%	(10)
Zhang et al.	2020.3.1–3.8	946	China (Ningbo)	Mixed healthcare workers	55.0%	(23)
Li et al.	2020.1.28–2.1	199	China (Wuhan)	Mixed frontline health professionals	34.2%	(24)
Liu et al.	2020.2.9–2.11	880	China	Mixed healthcare professionals	73.95%	(25)
Barello et al.	2020.4.4–4.27	532	Italy	Mixed healthcare professionals	41%	(26)
Duarte et al.	2020.5.9–6.8	2,008	Portugal	Mixed healthcare workers	53.1%	(27)
Matsuo et al.	2020.4.6–4.19	369	Japan	Mixed healthcare workers	31.4%	(28)
Agbobli et al.	2021.6.14–6.29	523	Togo	Mixed healthcare workers	53.5%	(29)
Akova et al.	2021.9.1–10.1	1015	Turkey	Mixed healthcare workers	56.7%	(30)
Medeiros et al.	2020.6–2020.7	265	Northeastern Brazil	Mixed healthcare workers	48.6%	(31)
Szwamel et al.	2020.6–2021.1	497	Poland	Mixed healthcare workers	71.63%	(32)

We found that 16.4% of the stay-behind medical staff suffered from severe job burnout, which indicated that some were suffering from a series of unhealthy states, such as depression, decreased sense of achievement and enthusiasm for work, as well as physical and mental exhaustion (1, 9). The persistence of such states can affect their physical and mental health and lead to a decline in their enthusiasm for work (9). This affects the implementation of various prevention and control measures during the normal prevention and control period of COVID-19, resulting in unpredictable consequences.

Night shift is one of the characteristics of medical jobs, which refers to the non-daily work hours. Some studies on its impact on the physical and mental health of medical staff found that working night shifts for a long term can induce many physiological and psychological diseases caused by the disorder of the circadian rhythm (11, 33). In this study, 87.6% of stay-behind medical staff had to work night shifts. Among them, 56.1% suffered from mild to moderate burnout, 17.4% suffered from severe burnout. While among those who did not work night shifts, it is 46.3 and 9.3%, respectively. And their differences were statistically significant. This may be because of the need to deal with all kinds of conditions independently during the night shift. The majority of patients at night need urgent care, which adds to the heavy work responsibilities. Studies have shown that the higher the frequency of night shifts, the more serious the burnout (11). According to a previous study, with the gradual increase of night shift duration, the burnout level and turnover intention of nurses also gradually increased. Considering the night shift duration of 8–9 h as the control, the turnover intention of those with the duration of 10–11 h, 12–13 h and more than 13 h were different. People with a night shift duration of more than 13 h have the highest level of job burnout and turnover intention (34), indicating that reasonable arrangement of duration is an important factor to manage job burnout.

Medical staff with job burnout will have anxiety, depression, and other negative emotions because of continuous work pressure. Meanwhile, emotional problems such as anxiety and depression are considered risk factors for job burnout (12). This study found that among the stay-behind medical staff, 63.8% accounted for non-depressed and 21.9, 13.1, and 1.2% accounted for mild, moderate, and severe depression, respectively. The overall incidence rate was 36.2%. Multinomial logistic regression analysis showed that depression was significantly associated with mild to moderate burnout and severe burnout, anxiety was associated with severe burnout. The severity of depression and anxiety among medical staff is closely related to job burnout, which is consistent with many studies (13, 35).

Social support, coping style, and burnout are important influencing factors of psychological stress (36). Coping style, and job burnout were risk factors of anxiety (37). Our study showed that better social support was associated with a lower incidence of job burnout among the stay-behind medical staff. We can speculate that targeted social support, such as increasing positive publicity, more psychological support, and a salary hike, can reduce the occurrence and development of job burnout and ensure the quality of medical treatment. It also showed that a positive coping style was significantly associated with a lower incidence of job burnout. Positive reinterpretation, an emotion-focused coping style, was a predictor of reduction of significant clinical anxiety (38), which is consistent with the results of our study. The guided application of positive coping styles may play a role in protecting the mental health of healthcare workers during their fight against the huge number of infectious diseases affecting society worldwide (38). Task-oriented coping was associated with decreased risk of burnout, while emotion-oriented coping was associated with increased risk (14). Coping style intervention may reduce burnout, while leading to improvement in medical staff’s wellbeing and patient outcomes.

Study had shown that living with one or more children at home was protective factor of burnout among healthcare workers (39). But in our study, we found that stay-behind healthcare workers with one child were more likely to suffer mild to moderate and severe burnout than those with two or more children. Further studies with large samples may be needed to clarify this issue.

A systematic review and meta-analysis of burnout among doctors show that professional title influenced burnout (40). It was based on 12 studies, including 6,320 doctors provided data pertaining to professional title (40). This is consistent with our findings: professional title were associated with severe burnout. This may be because stay-behind healthcare workers with junior and intermediate professional titles undertook most of the basic clinical work.

## Clinical perspectives

• The study evaluated burnout during the current Omicron wave of COVID-19 among stay-behind healthcare workers in Taizhou, China.
• In the prevention and control of COVID-19, some stay behind medical staff have been taking up a heavy load for a long time, further aggravating the risk of job burnout. To reduce the job burnout of stay-behind healthcare workers, hospital managers should focus on those who work night shifts as well as those who have depression tendencies, poor social support, one child, and non-positive coping styles. To reduce the severe burnout of stay-behind healthcare workers, hospital managers should also focus on those who have anxiety tendencies, junior and intermediate professional title. More rational shift schedules should be strictly enforced to reduce night shifts and workloads of frontline personnel. Targeted social support should be implemented, such as increasing positive publicity, providing more psychological support, and increasing salary, to reduce the occurrence and development of job burnout and ensure medical quality. Meanwhile, positive reinterpretation and task-oriented coping style intervention may reduce burnout, while leading to improvement in staff well-being and patient outcomes.

## Limitations

This study had several limitations. First, the data was obtained from a single tertiary hospital in China, and hence, findings may not apply to all stay-behind healthcare workers in China. Second, generalization requires a causal relationship. Third, the online data collection method is a limitation, which could potentially lead to over-reporting burnout among stay-behind healthcare workers. Fourth, our questionnaire did not account for all possible risk factors related to job burnout of stay-behind medical staff, such as the working hours, and salary; thus, the research results may be biased. Finally, the response rate was relatively low (25.0%), considering that the average age of the respondents was 34.13 ± 7.54 years, perhaps it is because some older medical staff are reluctant to participate in our questionnaire, as it is a relatively new thing in China. Since the survey respondents were likely to be younger than the general population of healthcare workers, it may result in selection bias.

## Conclusion

Job burnout among stay-behind medical workers was moderate during the current Omicron wave of COVID-19 in 2022. Night shift, depression, social support, positive coping, and number of children were associated with mild to moderate and severe burnout. Anxiety and professional title were associated with severe burnout. To alleviate burnout, government and hospital managers should consider intervening in these risk factors.

## Data availability statement

The raw data supporting the conclusions of this article will be made available by the authors, without undue reservation.

## Ethics statement

The studies involving human participants were reviewed and approved by the Ethics Committee of the Taizhou Hospital of Zhejiang Province (No. K20220410). Written informed consent for participation was not required for this study in accordance with the national legislation and the institutional requirements.

## Author contributions

T-HT, H-XC, M-XZ, and S-JP designed the study. S-JP, W-YQ, Y-PY, and X-MH collected the patient data. T-HT, W-YQ, and Y-PY analyzed and interpreted the patient data. S-JP, W-YQ, and T-HT wrote the manuscript together. All authors read and approved the final manuscript.
